# Cellular senescence mediates the detrimental effect of prenatal dexamethasone exposure on postnatal long bone growth in mouse offspring

**DOI:** 10.1186/s13287-020-01790-9

**Published:** 2020-07-06

**Authors:** Jianwen Su, Yu Chai, Zhiguo Ji, Yongheng Xie, Bin Yu, Xianrong Zhang

**Affiliations:** 1 Division of Orthopaedics and Traumatology, Department of Orthopaedics, Nanfang Hospital, Southern Medical University, No.1838 North of Guangzhou Avenue, Guangzhou, 510515 Guangdong Province China; 2 Guangdong Provincial Key Laboratory of Bone and Cartilage Regenerative Medicine, Nanfang Hospital, Southern Medical University, Guangzhou, 510515 China

**Keywords:** Dexamethasone, Bone development, Bone mesenchymal stem cells, Cellular senescence

## Abstract

**Background:**

Prenatal dexamethasone exposure (PDE) induces low birth weight and retardation of fetal bone development which are associated with lower peak bone mass in adult offspring. Here we evaluated whether and how PDE affects postnatal long bone growth in mouse offspring.

**Methods:**

Pregnant mice were injected subcutaneously with dexamethasone (1.2 mg/kg/day) every morning from gestational days (GD) 12–14. Femurs and tibias of 2-, 4-, 6-, and 12-week-old female offspring were harvested for histological, immunofluorescence, flow cytometric analysis, or microcomputed tomography (μCT) measurement.

**Results:**

PDE leads to impaired bone remodeling as well as decreased bone mass in the long bone of female mouse offspring. During postnatal bone growth, significant decrease of CD45^−^CD29^+^CD105^+^Sca-1^+^ bone marrow mesenchymal stem cells (BMSCs) and CD45^−^Nestin^+^ cells, loss of type H vessels, and increment of cellular senescence were found in metaphysis of long bone in mouse offspring after PDE. We further show that eliminating the excessive senescent cells with dasatinib (5 mg/kg/day) and quercetin (50 mg/kg/day) during GD 12–14 rescues the above toxic effect of PDE on the postnatal long bone growth in female mouse offspring.

**Conclusion:**

Cellular senescence mediates the toxic effect of PDE on postnatal long bone growth in mouse offspring, and inhibition of cellular senescence may be proposed for treating the retardation of bone growth caused by PDE.

## Introduction

Prenatal corticosteroid use in clinic has been shown to be effective in accelerating lung maturation and reducing the incidence of respiratory complications in infant [1]. However, clinical studies provide evidence that these short-term benefits are associated with reduction in birth size for infants born preterm, near term, or at term [2], and suppression of fetal bone turnover in infants at birth [3]. In addition, clinical trial data show that lower birth weight is associated with lower peak bone mass in adult offspring [4, 5]. Evidence from experimental animal models by our group and others demonstrate that prenatal dexamethasone exposure (PDE) impairs long bone development in fetal animals [6–9] and reduces bone mass in adult offspring [7, 10]. These data imply that developmental overexposure to glucocorticoid alters bone programming and results in less bone mass in offspring. Although studies have found the detrimental effect of PDE on osteogenesis [8, 11], little is known about the most critical cellular target of PDE in the developing bone.

Mesenchymal stem/stromal cells from bone marrow are multipotent cells that play crucial roles in bone development, maintenance, and regeneration due to their multilineage differentiation and self-renewal capacity [12, 13]. During childhood and puberty, skeletal bone grows quickly owing to rapid self-renewal and differentiation of bone marrow mesenchymal stem or stromal cells (BMSCs) and osteoprogenitors [14, 15]. The activities of stem cells are controlled by the local stem cell microenvironment, which is composed of cellular components such as stromal cells, immune cells, endothelial cells, osteoblasts, and a non-cellular compartment which includes extracellular matrix components and signal molecules [16]. This local environment plays important role in regulating stem cell survival, function, and fate [17], and alterations of the microenvironment may impair skeletal stem cells functions, leading to decreased osteogenesis and bone formation [18–20].

Cellular senescence is a state of irreversible growth arrest and occurs throughout life. Senescent cells can secrete numerous biologically active factors, termed the senescence-associated secretory phenotype (SASP), leading to pathological consequences in the tissue microenvironment [21]. It has been demonstrated that senescence associated bone microenvironment contribute to age-related bone loss [22]. Recent evidence suggests that cellular senescence is also a key regulator during bone development [23]. Senescence can be induced by various intrinsic and extrinsic triggers. Extensive use of dexamethasone has been associated with cellular senescence and aging-related pathological process in vivo and in vitro [24, 25]. However, the potential relationship between cellular senescence and development retardation in bone induced by PDE remains unclear.

Our recent study found that PDE during gestational days 12–14 (GD 12–14) retards bone development in fetal mice [6]. Here we show that young and adult female mouse offspring by PDE have impaired bone formation and significant lower bone mass. We have found significantly reduced amount of CD45^−^Nestin^+^ and CD45^−^CD29^+^CD105^+^Sca-1^+^ cells as well as impaired angiogenesis in metaphysis of long bone in young offspring mice after PDE. We also have found that PDE promotes cellular senescence and suppresses cells proliferation in trabecular area of long bone. Interestingly, eliminating senescent cells using senolytics dasatinib and quercetin (D+Q) rescues significantly the decreased BMSCs and osteoprogenitors by PDE and prevents developmental retardation of long bone in young mouse offspring.

## Materials and methods

### Animals

This study was conducted in accordance with the Guide for the Care and Use of Laboratory Animals of Nanfang Hospital Southern Medical University. The protocol was approved by the Animal Care and Use Committee of Nanfang Hospital. Pathogen-free mice were maintained under standard conditions in a 12-h light and 12-h dark cycle, at 25 ± 3 °C, with a relative humidity of 40–60%, and with food and tap water available ad libitum. PDE during GD 12–14 was applied according to the procedure previously described [6]. Briefly, virgin C57BL/6 female mice at 10–12 weeks old were mate with male mice overnight. The day on which the presence of a vaginal plug was set as GD 0, the pregnant mice were randomly assigned to the PDE group or vehicle treatment (control) group. To construct PDE mice model, dexamethasone sodium phosphate (Cat. 2392-39-4, Tianxin, China) was injected subcutaneously (1.2 mg/kg/day) during GD 12–14. To construct the vehicle control of PDE model, pregnant mice were treated with the same amount of vehicle (normal saline) daily during GD 12–14. The pregnant mice were housed individually in cages with freely available food and water. Two female offspring were selected randomly from each litter for postnatal bone development investigation. Femurs and tibias from mouse offspring at 2-, 4-, 6-, and 12-week-old were dissected for further analysis. To evaluate the effect of dasatinib (S1021, Selleck Chemicals, Houston, TX, USA) and quercetin (S2391, Selleck Chemicals, Houston, TX, USA), PDE pregnant mice during GD 12–14 were treated with vehicle (200 μl 1% methyl cellulose) or dasatinib (5 mg/kg/day) plus quercetin (50 mg/kg/day) by oral gavage, respectively.

### Microcomputed tomography (μCT) analysis

Tibias from 12-week-old mouse offspring were dissected free of soft tissue, fixed and stored in 70% ethanol, and imaged using a μCT specimen scanner (Scanco Medical, AG, Switzerland). The scan was performed using an X-ray energy of 55 kV and current of 145 mA, with a voxel size of 12 μm and an integration time of 400 ms. Trabecular bone measurements consisting of 250 slices (3 mm) were performed from 0.215 mm (18 image slices) below the growth plate. Bone volume (BV/TV), trabecular number (Tb. N), trabecular thickness (Tb. Th), and trabecular separation (Tb. Sp) were determined. Quantitative analyses were carried out using IPL software (Image Processing Language V5.15, Scanco Medical AG, Switzerland).

### Histochemistry

To study the morphology of postnatal long bone growth in offspring after PDE, femurs and tibias of mouse offspring at 2-, 4-, and 6-week-old were fixed in 4% paraformaldehyde, decalcified in 0.5 M ethylenediamine-tetraacetic acid (EDTA, pH 7.4), followed by paraffin embedding or frozen embedding. Hematoxylin-eosin (H&E) staining, Goldner’s trychrome staining, and tartrate-resistant acid phosphatase (TRAP) staining were performed on 4-μm paraffin sections according to standard procedures. The number of osteoblasts per square millimeter of metaphyseal area (N. per mm^2^) was quantified in the area from 0 to 0.5 mm below growth plate.

For detecting the osteoclastic activity in bone, TRAP staining was performed on the deparaffinized and rehydrated sections using a Leukocyte Acid Phosphatase kit (Cat. 387A-1KT, Sigma-Aldrich, USA). The TRAP^+^ multinucleated cells containing at least three nuclei were identified as osteoclasts under light microscope (Olympus, BX53). The number of TRAP^+^ cells per square millimeter of metaphyseal area (N. per mm^2^) in the area from 0 to 0.5 mm below growth plate was quantified.

For detecting senescence-associated β-galactosidase (SA-β-Gal) activity, frozen sections were stained using SA-β-Gal staining kit (Cat. 9860, Cell Signaling Technology, USA) according to the manufacturer’s instructions. Senescent cells were identified as blue-stained cells under light microscope (Olympus, BX53). The number of SA-β-Gal^+^ cells per square millimeter of metaphyseal area (N. per mm^2^) in the area from 0 to 0.5 mm below growth plate was quantified.

### Immunofluorescence

For immunofluorescence staining, frozen sections were incubated in blocking buffer (3% BSA in PBS with Tween (PBST)) for 1 h at room temperature, incubated with primary antibodies overnight at 4 °C. The primary antibodies for immunostaining include the following: Nestin (ab134017, Abcam, Cambridge, MA, USA), CD31 (FAB3629G-100, R&D Systems, Minneapolis, MN, USA), Endomucin (Emcn, SC-65495, Santa Cruz, Dallas, TX, USA), and Ki67 (ab15580, Abcam, Cambridge, MA, USA). Sections were washed 3 times in PBS and then incubated with secondary antibodies at room temperature for 1 h. The secondary antibodies for immunostaining include the following: 488-conjugated secondary antibody (703-546-155, Jackson ImmunoResearch, West Grove, PA, USA), 594-conjugated secondary antibody (712-586-153, Jackson ImmunoResearch, West Grove, PA, USA), and 488-conjugated secondary antibody (A21206, ThermoFish Scientific, USA). Nuclei were counterstained with DAPI (S2110, Solarbio, China). Images were captured using a fluorescence microscope (Olympus, BX53, Japan). Positive-stained area or the number of positive-stained cells per square millimeter of the metaphyseal area was measured from 0 to 0.5 mm below growth plate.

### Flow cytometric analysis

Bone marrow cells were collected from femurs and tibias of mouse offspring at 4-week-old. Cell numbers were determined after removal of red blood cells with ACK Lysis Buffer (CS0001, Leagene, China). After being washed with PBS twice, pellets were resuspended and blocked in 1% BSA on ice for 15 min. Cells were then washed twice with PBS and incubated with primary antibody (for cell surface marker) solution diluted by 0.5% BSA for 30 min on ice in the dark. After being fixed by 4% paraformaldehyde and permeabilized by PBST and washed with PBS, cells were incubated with primary antibody (for intracellular antigen) solution diluted by 0.5% BSA for 30 min on ice in the dark. The primary antibody used were PE-conjugated Nestin Antibody (MA5-23574, ThermoFish Scientific, Rockford, IL, USA) and BV421-conjugated anti-mouse CD45 (563890, BD Biosciences, San Jose, CA, USA). Cells were then washed once and re-suspended in 300 μl PBS and transferred to flow tubes. For BMSCs, CD45^−^CD29^+^CD105^+^Sca-1^+^ bone marrow cells were detected using a Mouse mesenchymal stem cell Multi-color Flow kit (FMC003, R&D systems, Canada) following the manufacturer’s protocol. Flow cytometric analysis was performed on a BD LSRFortessa flow cytometer (BD Biosciences, San Jose, CA, USA) and analyzed using FlowJo software (BD Life Sciences San Jose, CA, USA).

### Statistics

The data and statistical analysis comply with the recommendations on experimental design and analysis in pharmacology. All quantitative data were presented as mean ± S.E.M. For comparisons between two groups, independent Student’s *t* test was performed. For multiple comparisons, one-way analysis of variance (ANOVA) with Bonferroni post hoc test was used. Statistical analysis was performed using SPSS, version 20 software (International Business Machines Corporation, IBM Corp.). Significant level was defined as *P* < 0.05.

## Results

### Adult mouse offspring from PDE has low bone mass in the long bone

**Fig. 1:**
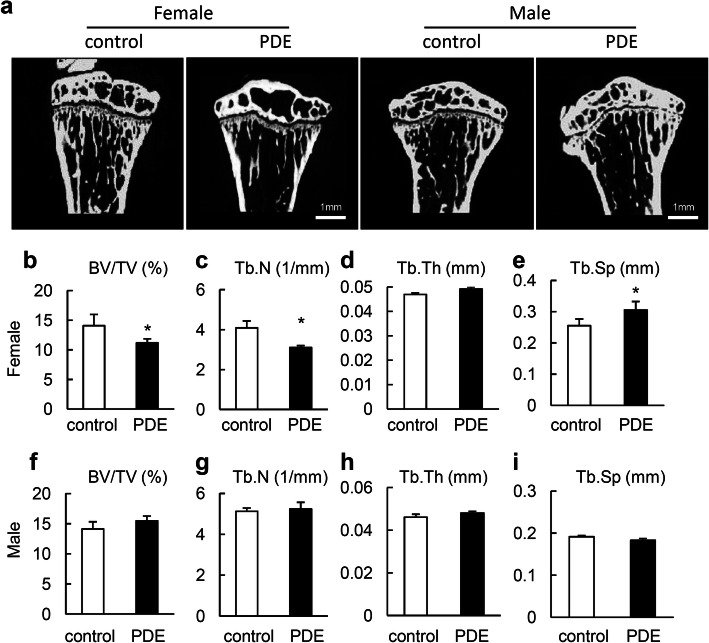
Prenatal dexamethasone exposure (PDE) induces bone loss in later life of female mouse offspring. **a** Representative μCT images of proximal tibia in 12-week-old female and male mouse offspring. Scale bar, 1 mm. Quantitative analyses of tibial microstructural parameters of female mouse offspring including trabecular bone volume fraction (BV/TV) (**b**), trabecular number (Tb. N) (**c**), trabecular thickness (Tb. Th) (**d**), and trabecular separation (Tb. Sp) (**e**). Quantitative analysis of BV/TV (**f**), Tb. N (**g**), Tb. Th (**h**), and Tb. Sp (**i**) in tibia of male mouse offspring. Data are represented as mean ± S.E.M. *P < 0.05 versus control (*n* = 6 per group, Student’s *t* test)

Our previous work has shown the adverse effect of PDE on the long bone development in fetal mice [6]. We then tested whether long bone development retardation during prenatal period results in bone mass deficit in adult offspring. Tibias were harvested from 12-week-old mouse offspring of PDE and measured by μCT. A significant reduction in the mass of trabecular bone was observed in female offspring relative to control mouse offspring (Fig. 1). There was a significant decrease of bone volume fraction (BV/TV) in female adult mouse offspring from PDE, which was attributed to a large decrease in trabecular number (Tb. N) along with a notable increase in trabecular separation (Tb. Sp), with no change observed in trabecular thickness (Tb.Th) (Fig. 1b–e). But, we did not observe change of the above microstructural parameters in male mouse offspring (Fig. 1f–i). Together, these data revealed that PDE has long-term detrimental effect on bone mass in female adult mouse offspring.

### Postnatal long bone development is retarded in female mouse offspring from PDE

**Fig. 2:**
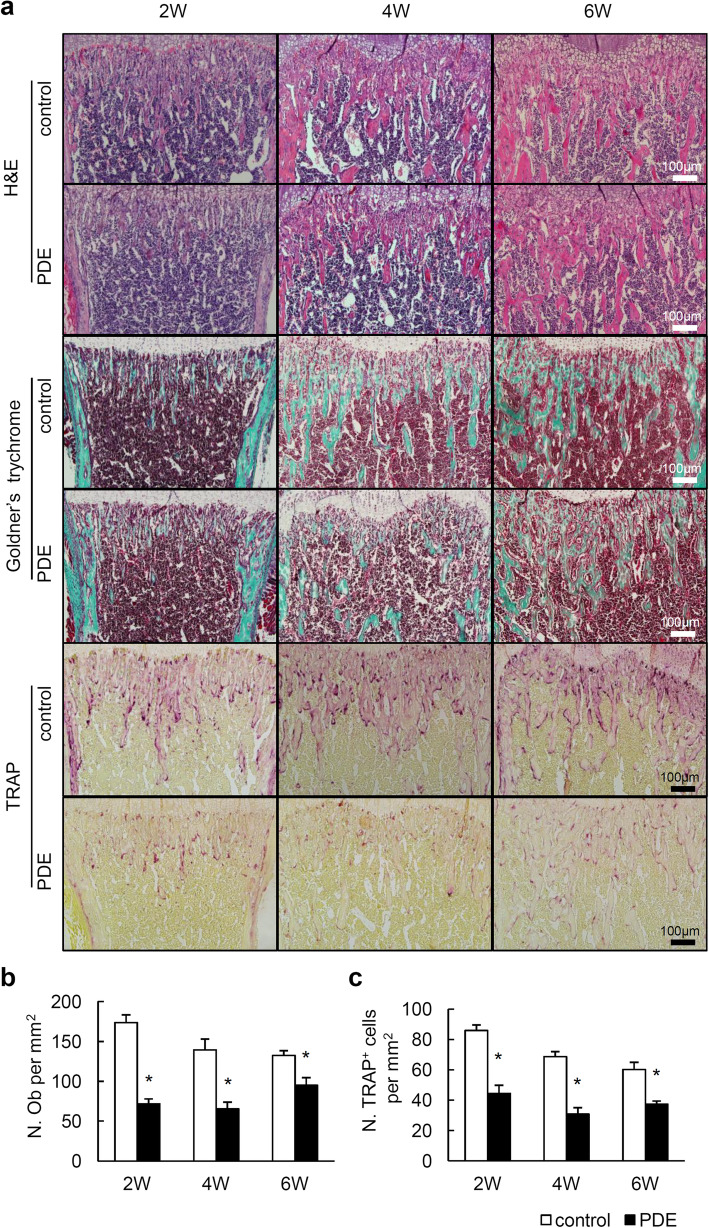
Prenatal dexamethasone exposure (PDE) suppresses postnatal long bone development in female mouse offspring. **a** Representative images of hematoxylin-eosin (H&E) staining, Goldner’s trychrome staining, and tartrate-resistant acid phosphatase (TRAP) staining in femoral sections from 2-, 4-, and 6-week-old female mouse offspring. 2W, 4W, and 6W represent 2-, 4-, and 6-week-old mice, respectively. Scale bar, 100 μm. Quantitative analysis of the numbers of osteoblast per tissue area in metaphyseal bone below growth plate (N. Ob per mm^2^) (**b**) and the numbers of TRAP^+^ cells per tissue area in metaphyseal bone below growth plate (N. TRAP^+^ cells per mm^2^) (**c**). Data are represented as mean ± S.E.M. *P < 0.05 versus control (*n* = 5 per group, Student’s *t* test)

Bone mass in adulthood has been closely correlated with fetal and postnatal bone growth, which is an important process for bone mineral accrual [26]. To investigate how PDE induces low bone mass in female adult offspring, we evaluated the morphology of long bone of female offspring at 2-, 4-, and 6-week-old. H&E staining and Goldner’s trychrome staining results showed reduced amount of trabecular bones in female mouse offspring after PDE (Fig. 2a). Similarly, histomorphometric analysis revealed significantly less osteoblast counts in offspring from PDE, compared with that in control (Fig. 2b). As the reduced trabecular bone could be due to either lower bone formation or higher bone resorption or both, we examined the changes of osteoclastogenesis in mouse offspring after PDE. TRAP staining and quantitative analysis revealed that osteoclast number was significantly lower in PDE mouse offspring compared to controls (Fig. 2c). Therefore, PDE impedes both bone formation and bone absorption during skeletal bone development.

### PDE induces loss of Nestin expressing cells and blood vessels in long bone of female mouse offspring

**Fig. 3:**
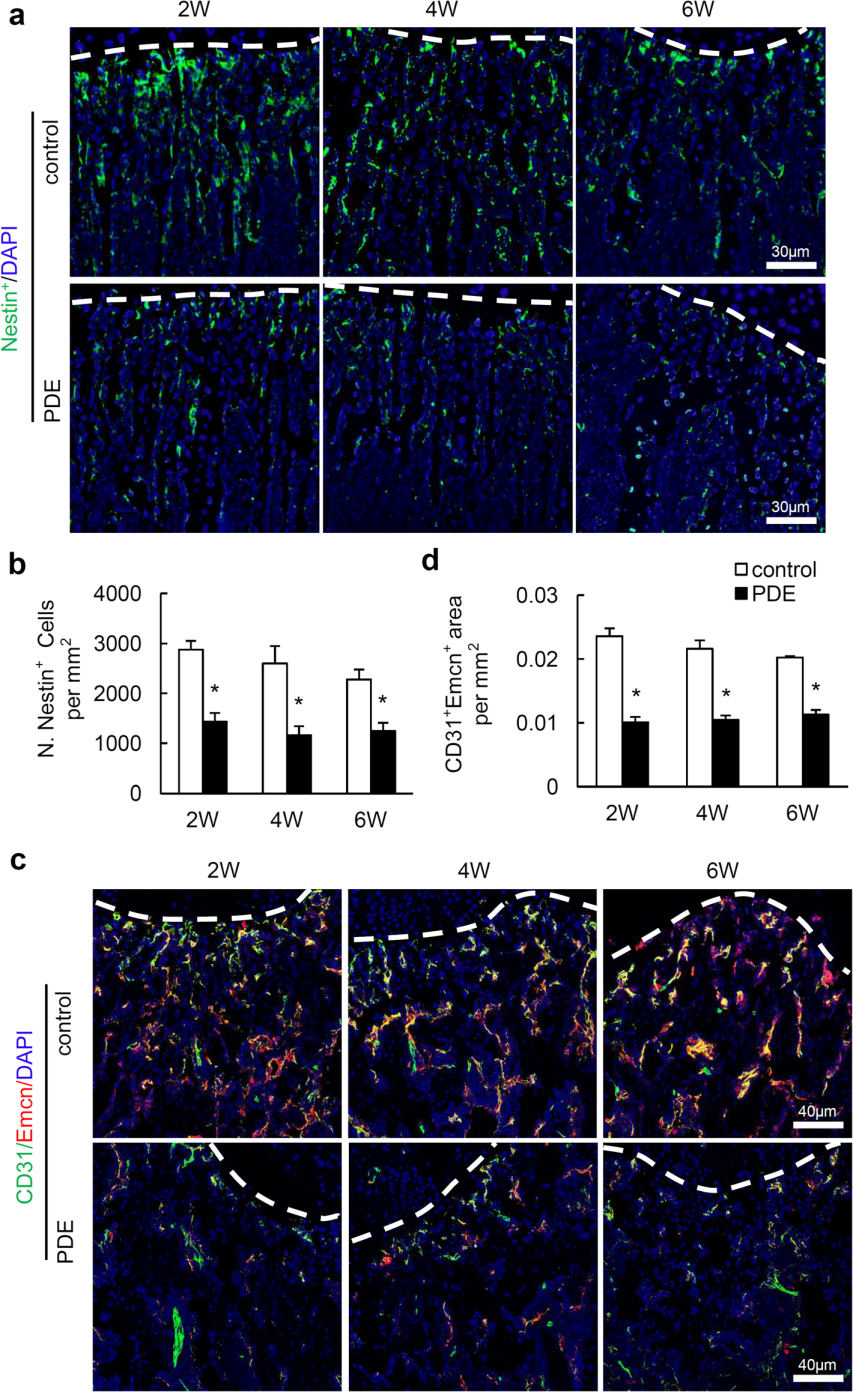
Prenatal dexamethasone exposure (PDE) induces loss of Nestin^+^ cells and type H vessels in the long bone of female mouse offspring. **a** Representative images of immunofluorescence staining for Nestin (green) and **b** quantitative analysis of Nestin^+^ cells in femoral metaphysis from 2-, 4-, and 6-week-old female offspring. 2W, 4W, and 6W represent 2-, 4-, and 6-week-old mice, respectively. DAPI stains nuclei blue. Scale bar, 30 μm. **c** Representative images of double-immunofluorescence staining for CD31 (green) and Endomucin (Emcn, red) in femoral metaphysis from 2-, 4-, and 6-week-old female offspring. DAPI stains nuclei blue. Scale bar, 40 μm. **d** Quantification of the relative fluorescence area of CD31^+^Emcn^+^ cells per tissue area in femoral metaphysis (CD31^+^Emcn^+^ area per mm^2^). Data are represented as mean ± S.E.M. *P < 0.05 versus control (*n* = 5 per group, Student’s *t* test)

Nestin expressing (Nestin^+^ cells) in postnatal bones are heterogeneous populations mainly in endothelial and osteoblast lineage [23, 27]. These cells are highly proliferative and critical for osteoblast replenishment for bone formation during postnatal bone development [23]. We thus assessed whether PDE affects Nestin^+^ cells in postnatal long bone of female mouse offspring. Results showed that the number of Nestin^+^ cells in femoral metaphysis was significantly reduced as assessed by immunofluorescence staining (Fig. 3a, b).

A specific subtype of vessels, termed H-type vessels which characterized by high expression of the endothelial markers CD31 and Emcn (CD31^hi^Emcn^hi^), generate distinct microenvironments for maintaining perivascular osteoprogenitors and coupling angiogenesis to osteoprogenitors [28]. We then examined the effect of PDE on type-H vessels in postnatal long bone of offspring. Double immunofluorescence staining for CD31 and Emcn showed a significant lower proportion of type-H vessels in the femoral metaphysis of mouse offspring after PDE compared to that of control offspring (Fig. 3c, d). Therefore, PDE reduces type-H vessels, which are closely associated with impaired bone formation in mouse offspring.

### Cellular senescence is increased in postnatal long bone of mouse offspring after PDE

**Fig. 4:**
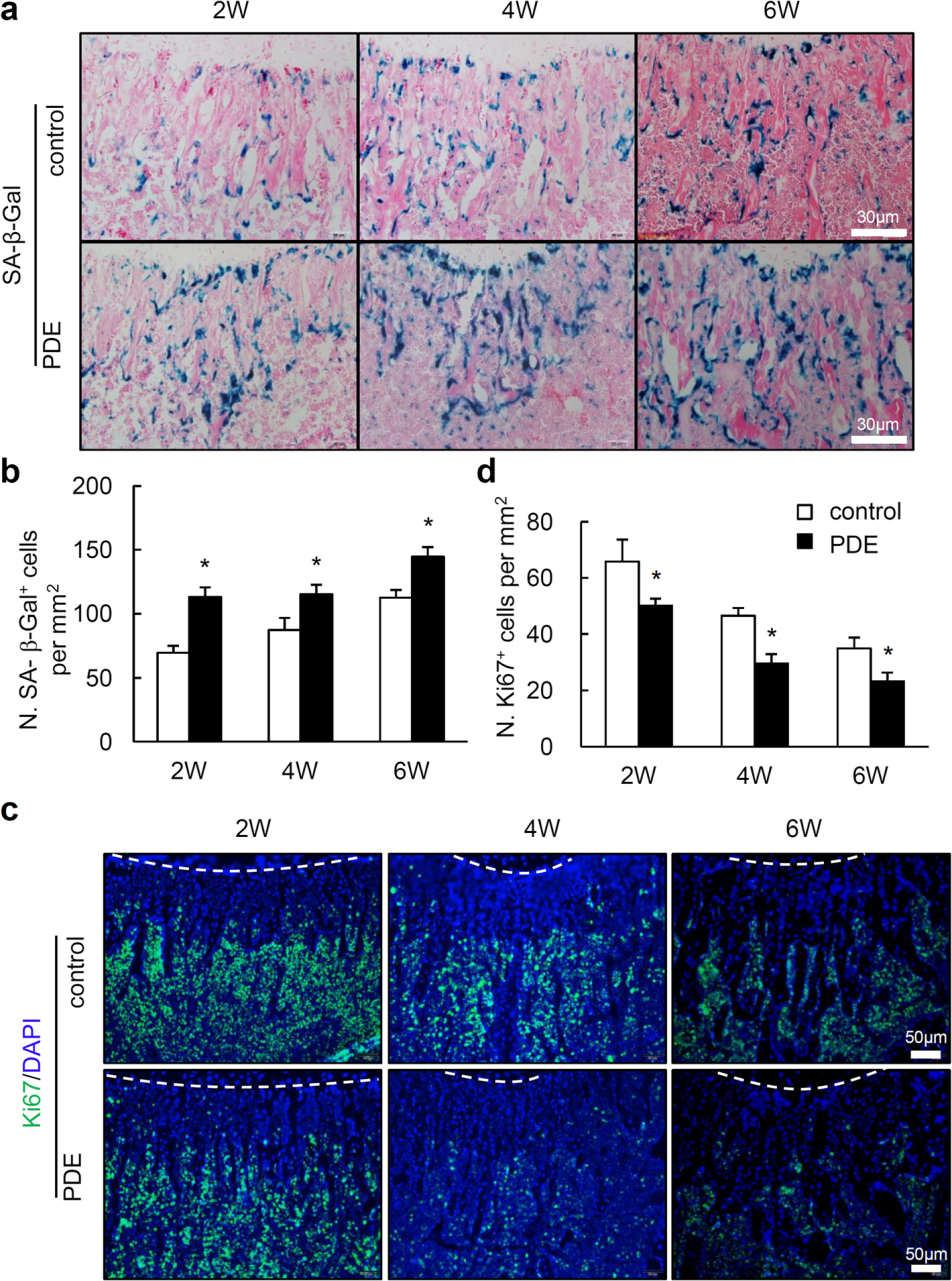
Prenatal dexamethasone exposure (PDE) enhances cellular senescence in trabecular bone of female mouse offspring. **a** Representative images of senescence associated β-galactosidase (SA-β-Gal) staining in femoral metaphysis from 2-, 4-, and 6-week-old female offspring. 2W, 4W, and 6W represent 2-, 4-, and 6-week-old mice, respectively. Sections were counterstained with eosin (pink color). Scale bar, 30 μm. **b** Quantitative analysis of the number of SA-β-Gal^+^ cells per tissue area in metaphyseal bone below growth plate. **c** Representative images of immunofluorescence staining for Ki67 (green) and **d** quantitative analysis of the number of Ki67^+^ cells in femoral metaphysis from 2-, 4-, and 6-week-old female offspring. DAPI stains nuclei blue. Scale bar, 50 μm. Data are represented as mean ± S.E.M. *P < 0.05 versus control (*n* = 7 per group, Student’s *t* test)

A recent report showed that premature cellular senescence in the long bone of young mice leads to reduction of osteoprogenitors, impairing blood vessel formation and bone formation [23]. We then tested whether the progression of the cellular senescence in long bone of postnatal offspring is altered by PDE. SA-β-Gal staining was conducted in femoral bones of mouse offspring at 2-, 4-, and 6-week-old. We found a significant increase in the number of SA-β-Gal^+^ cells in femoral metaphysis of PDE offspring at 2-, 4-, and 6-week-old compared to those in control offspring, respectively (Fig. 4a, b). Consistently, immunofluorescence staining for Ki67, the proliferative marker, showed reduced staining in the trabecular bone adjacent to the growth plate, the same region that SA-β-Gal^+^ cells located (Fig. 4c). Quantitative results confirmed dramatically reduction of Ki67^+^ cells in the femoral metaphysis of mouse offspring after PDE (Fig. 4d). The above results strongly suggest that PDE induces growth arrest and senescence in certain cell types. To identify whether PDE induced senescence in osteoprogenitors, we performed co-staining of SA-β-Gal with osteoprogenitor marker Nestin and Osterix, and vessel marker Emcn, respectively. However, we did not find overlapping staining of SA-β-Gal with any of those three markers (Supplementary Fig. 1, 2). The above data suggest that PDE might not stimulate cellular senescence in osteoprogenitors and cells of type H vessels directly, but suppress those cells indirectly by stimulating senescence of other cell populations in bone.

### Targeting cellular senescence prevents PDE-induced bone development retardation in mouse offspring

**Fig. 5:**
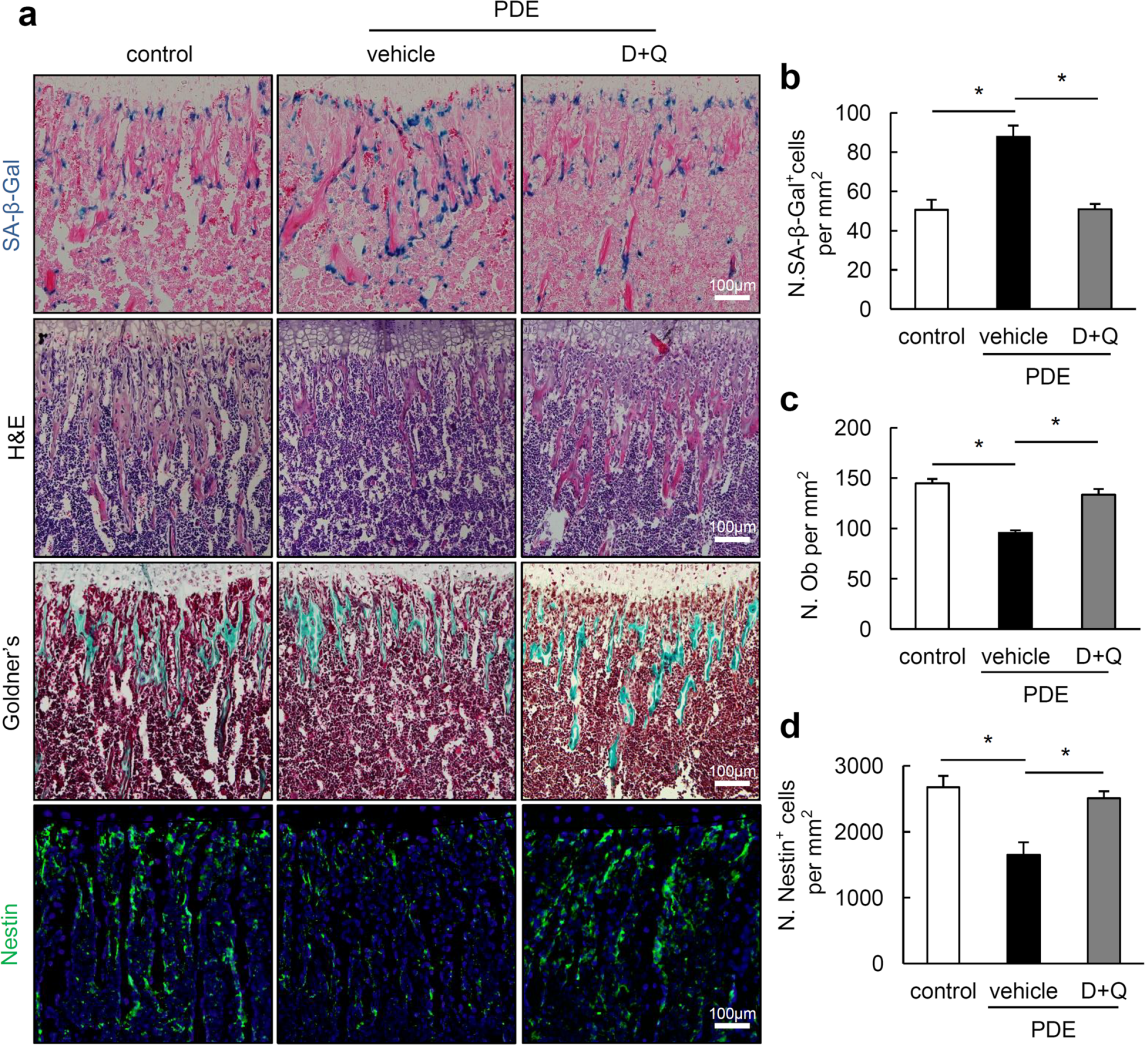
Clearance of senescent-cell by treatment with senolytics dasatinib and quercetin (D+Q) rescues the suppressed osteogenesis in female mouse offspring after PDE. **a** Representative images of senescence associated β-galactosidase (SA-β-Gal) staining, hematoxylin-eosin (H&E) staining, Goldner’s trychrome staining, and Nestin immunofluorescence (green) in femoral metaphysis from 2-week-old female offspring. Pregnant mice were injected subcutaneously with normal saline (control) or dexamethasone (1.2 mg/kg/day) (PDE group) during gestational days (GD) 12–14. Additionally, control pregnant mice were treated with 1% methyl cellulose, and PDE pregnant mice were treated with 1% methyl cellulose (vehicle) or dasatinib (5 mg/kg/day) and quercetin (50 mg/kg/day) by oral gavage during GD 12–14. Femurs of 2-week-old female mouse offspring were harvested for analysis. DAPI stains nuclei blue. Scale bar, 100 μm. **b** Quantitative analysis of the numbers of SA-β-Gal^+^ cells per tissue area in femoral metaphysis. *n* = 6 per group. **c** Quantitative analysis of the osteoblast numbers per tissue area in femoral metaphysis. *n* = 6 per group. **d** Quantitative analysis of the Nestin^+^ cells per tissue area. *n* = 5 per group. *P < 0.05, one-way analysis of variance (ANOVA) with Bonferroni post hoc test

It was reported that clearance of senescent cells using D+Q can improve bone mass in aged mice [22]. To evaluate the role of increased cellular senescence in PDE-induced bone growth retardation in mouse offspring, pregnant mice by PDE were treated with D+Q or vehicle during GD 12–14 once every day. Consistent with the effect of D+Q on aged mice, SA-β-Gal^+^ cells number in femoral metaphysis was significantly suppressed in D+Q-treated PDE mouse offspring as compared to vehicle-treated ones (Fig. 5a, b). As anticipated, D+Q treatment significantly improved trabecular bone amount in femoral metaphysis in 2-week-old PDE mouse offspring relative to vehicle-treated ones, as assessed by H&E staining and Goldner’s trychrome staining results (Fig. 5a). Trabecular bone histomorphometry demonstrated the rescued osteoblast numbers and increased Nestin^+^ cells in PDE mouse offspring treated by D+Q than in vehicle-treated ones (Fig. 5c, d).

**Fig. 6:**
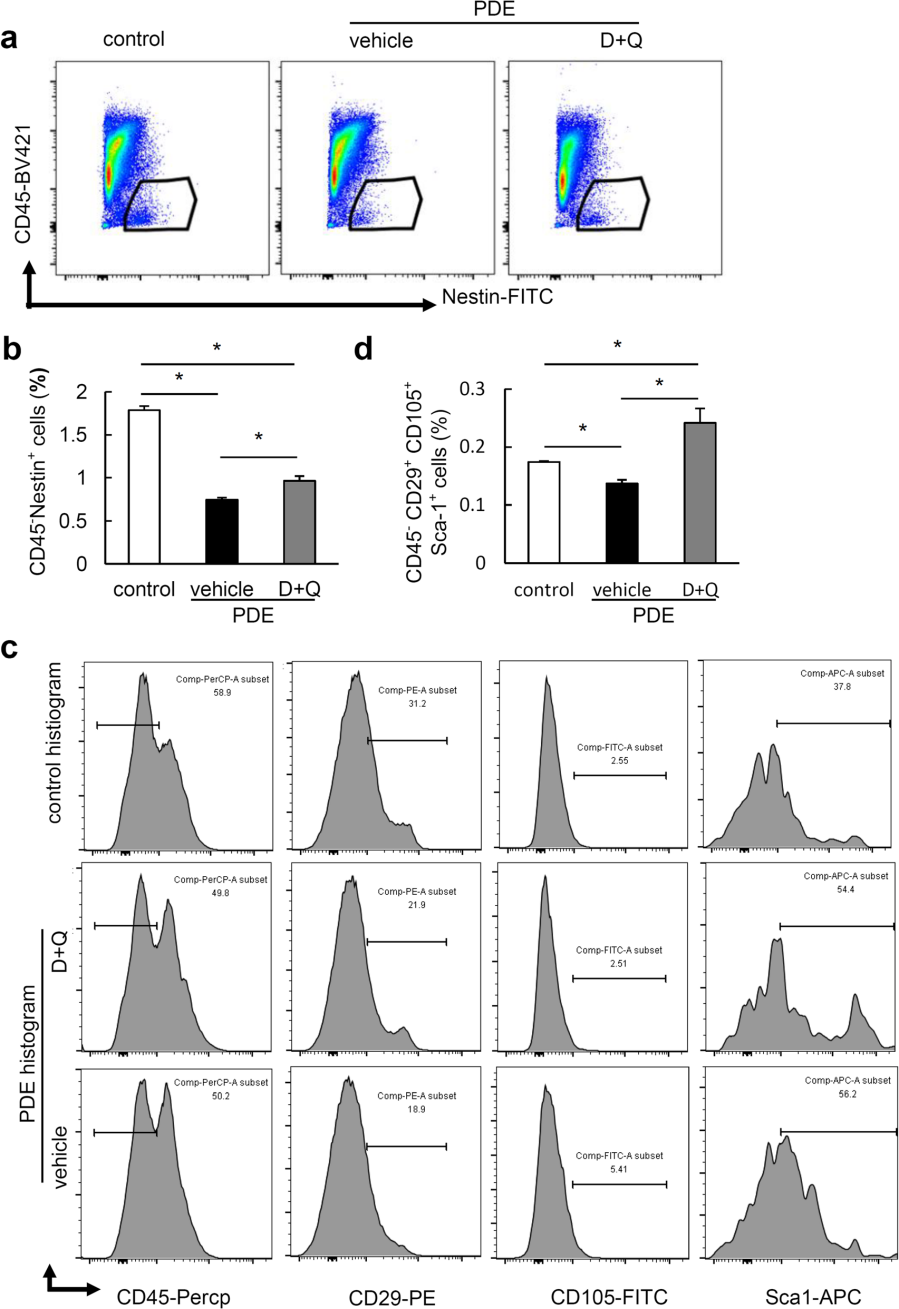
Clearance of senescent cell by treatment with senolytics dasatinib and quercetin (D+Q) rescues the loss of osteoprogenitors and BMSCs in female mouse offspring after PDE. **a** Representative images of the flow cytometry analysis and **b** the percentage of Nestin^+^ cells in femoral bone from 4-week-old female mouse offspring. Pregnant mice were injected subcutaneously with normal saline (control) or dexamethasone (1.2 mg/kg/day) (PDE group) during gestational days (GD) 12–14. Additionally, control pregnant mice were treated with 1% methyl cellulose, and PDE pregnant mice were treated with 1% methyl cellulose (vehicle) or dasatinib (5 mg/kg/day) and quercetin (50 mg/kg/day) by oral gavage during GD 12–14. Femoral bone marrow of 4-week-old female mouse offspring was harvested for analysis. **c** Representative images of the flow cytometry analysis and **b** the percentage of CD45^−^CD29^+^CD105^+^Sca-1^+^ cells in femoral bone from 4-week old female mouse offspring. *n* = 5 per group. *P < 0.05, one-way analysis of variance (ANOVA) with the Bonferroni post hoc test

**Fig. 7:**
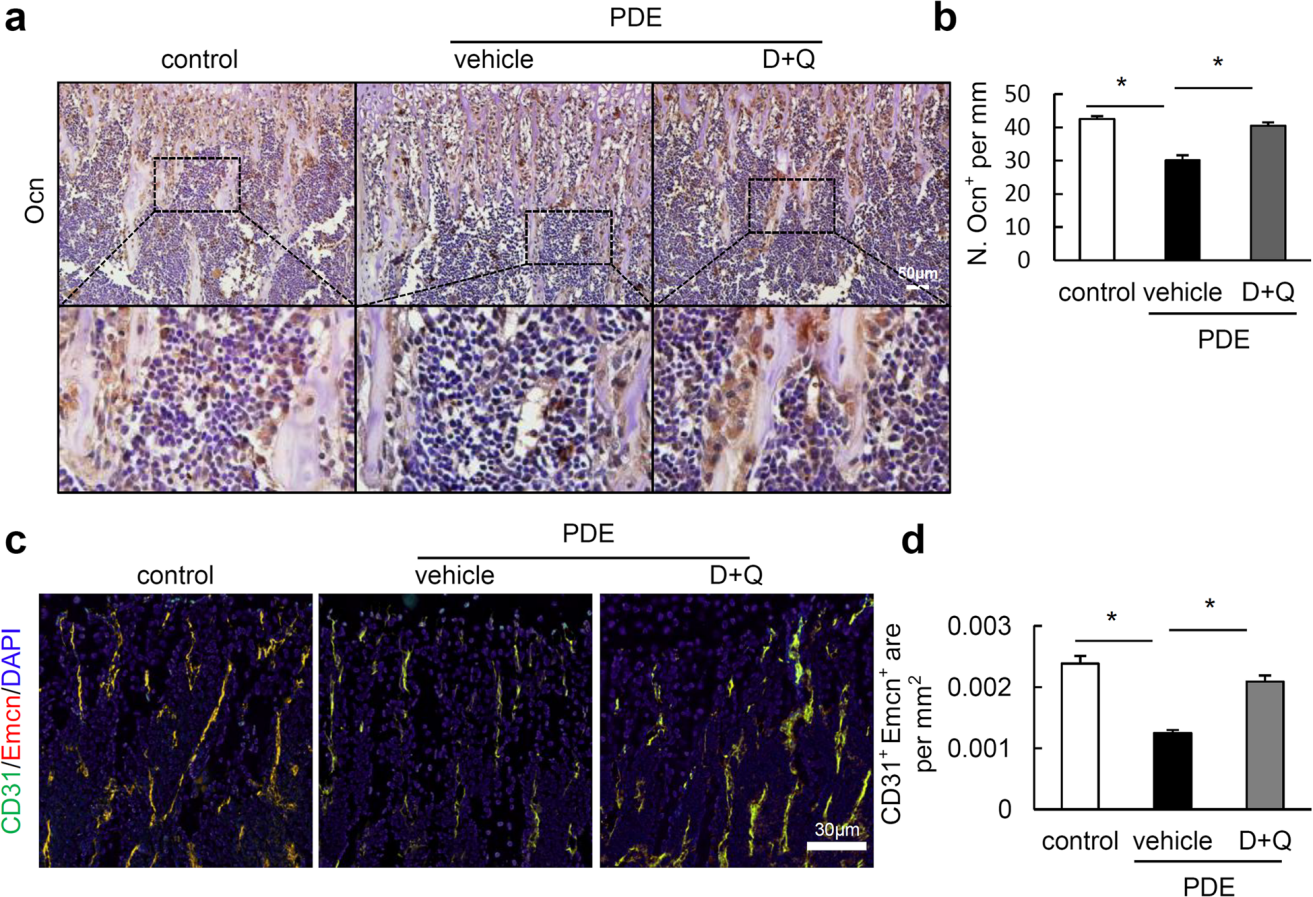
Senolytics dasatinib and quercetin (D+Q) rescues the loss of osteoblasts and H type vessels in female mouse offspring after PDE. **a** Representative images of the immunohistochemistry staining for osteocalcin (Ocn, dark brown stained cells on the surface of trabecular bone) and **b** the number of Ocn^+^ cells per mm of bone surface in femoral bone from 4-week-old female mouse offspring. Scale bar, 50 μm. **c** Representative images of double-immunofluorescence staining for CD31 (green) and Endomucin (Emcn, red) in femoral metaphysis from 4-week-old female offspring. DAPI stains nuclei blue. Scale bar, 30 μm. **d** Quantification of the relative fluorescence area of CD31^+^Emcn^+^ cells per tissue area in femoral metaphysis (CD31^+^Emcn^+^ area per mm^2^). Data are represented as mean ± S.E.M. *n* = 5 per group, *P < 0.05, one-way analysis of variance (ANOVA) with the Bonferroni post hoc test

Concomitant with reduced burden of cellular senescence by D+Q and markedly rescued bone phenotype in mouse offspring after PDE, we further found that the percentage of CD45^−^Nestin^+^ (Fig. 6a, b) and CD45^−^CD29^+^CD105^+^Sca-1^+^ (Fig. 6c, d) was significantly decreased in bone marrow of mouse offspring after PDE. It is noteworthy that D+Q treatment only partially rescued the loss of CD45^−^Nestin^+^ cells in mouse offspring after PDE, but completely restored the ratio of CD45^−^CD29^+^CD105^+^Sca-1^+^ cells (Fig. 6). Compared with control mice, mouse offspring after PDE and D+Q treatment had significantly lower percentage of CD45^−^Nestin^+^ cells (Fig. 6a, b), but had a considerable increment in the percentage of CD45^−^CD29^+^CD105^+^Sca-1^+^ cells (Fig. 6b, d). Additionally, D+Q treatment also dramatically restored the osteoblast number (Fig. 7a, b) and angiogenesis potential in mouse offspring after PDE (Fig. 7c, d). All the data indicate that cellular senescence mediates the detrimental effect of PDE on BMSCs and osteoprogenitors, leading to retardation of postnatal long bone growth in mouse offspring.

## Discussion

Here we provide unique insights into the mechanisms by which PDE leads to retardation of postnatal bone development. Our data show that PDE induces cellular senescence, reduction of BMSCs amount, and decrease of bone modeling and remodeling during postnatal bone growth in female mouse offspring. Furthermore, elimination of excessive senescent-cells can rescue the toxic effect of PDE on postnatal bone growth in female mouse offspring. The present study has identified that PDE induces cellular senescence which may contribute to the toxic effect of PDE on BMSCs and type H vessels in the metaphysis of long bone, thereby impeding bone formation in mouse offspring. We believe that these results have opened a door to elucidating poorly understood aspects of osteoporosis with developmental origin and highlighted D+Q as a therapeutic approach for treating PDE-induced retardation of bone growth in offspring.

Our previous work demonstrated that continuous PDE during GD 9–20 retards long bone development in fetal rats and leads to low bone mass in adult rat offspring [7], and further delineated that PDE at dose higher than 0.8 mg/kg/day during GD 12–14 developed severe retardation of long bone development in fetal mice [6]. In the present study, the data clearly indicate that PDE at 1.2 mg/kg/day during GD 12–14 induces low bone mass in 12-week-old mouse offspring. Our finding is supported by a recent report that PDE at GD 16–17 induces decreased bone mass and biomechanical strength in adult mouse offspring [29]. Furthermore, we observed a notable decrease in both osteoblasts and osteoclasts in femoral metaphysis of female mouse offspring after PDE, indicating a decreased bone modeling and remodeling induced by PDE. Overall, the works from our lab and others have highlighted that PDE has long-term effect on bone mass of offspring in later life, which might be consequences of PDE programming on intrauterine development of fetal tissue [30, 31].

During bone modeling and remodeling, bone formation requires continuous production of osteoblasts from osteoprogenitors and BMSCs in mammals. Nestin^+^ cells in postnatal bone encompass osteoblastic and endothelial progenitors, and several subsets of stem cells with self-renewal capacity [27, 32]. Additionally, type H vessels are important for maintaining osteoprogenitors and coupling angiogenesis to osteoprogenitors [28]. Our studies provide the first evidence of decreased amount of CD45^−^CD29^+^CD105^+^Sca-1^+^ BMSCs and Nestin^+^ cells and impaired type H vessels in the long bone of female mouse offspring after PDE. It is possible that PDE has extensive toxic effect on osteoprogenitors, BMSCs, and type H vessels, leading to impaired osteogenesis and diminished bone formation.

Interestingly, we found the dramatically increased cellular senescence and diminished cellular proliferation in femoral metaphysis of young female mouse offspring after PDE. Senescent cells are known to instigate SASP which may exert detrimental paracrine, thereby contributing to senescence-related inflammation and stem cell dysfunction [33]. Indeed, senescent cells are found to be associated with embryonic and postnatal development [23, 34, 35]. In the present study, we did not find much overlapping of osteoprogenitor markers with SA-β-Gal staining; however, our data revealed that reducing senescent cell burden using senolytics D+Q has dramatically restored the percentage of BMSCs, the numbers of osteoblasts, and type H vessels in trabecular bone of female mouse offspring after PDE. These data support our conclusion that excessive senescent-cells mediate the detrimental effect of PDE on postnatal long bone development in female mouse offspring.

As Nestin^+^ cells has been found to undergo normal programmed senescence in trabecular bone during late puberty, and prednisolone injection during puberty further stimulate senescence of Nestin^+^ cells in mice [23], it is possible that loss of Nestin^+^ cells in PDE mouse offspring might be at least partially due to the senescence of Nestin^+^ cells. Interestingly, we found that senolytics D+Q treatment only partially rescued the loss of Nestin^+^ cells in mouse offspring after PDE. One reason could be that PDE suppresses Nestin^+^ cells by a mechanism of action that is distinct from that of D+Q, which is known to reduce senescent cell burden by reducing pro-inflammatory cytokine secretion [36].

Bone is a dynamic organ composed of various bone marrow-derived cell types including hematopoietic, mesenchymal, endothelial cells [37]. As dexamethasone has far-ranging effects on various cells and tissues [30], it might trigger the senescence on a heterogeneous population of cells in bone. Considering that almost all of SA-β-Gal-positive stained cells locate in endosteal bone marrow close to the trabecular bone surface, but not inside the trabecular bone where osteocytes locate, it is reasonable to rule out the senescence of osteocytes in young mouse offspring after PDE. Nevertheless, a limitation of our study cannot be neglected that we failed (despite extensive effort) to identify the cell types and molecular mechanism of cellular senescence induced by PDE. Growing evidences indicate that prenatal glucocorticoid may exert long-term programming of fetal tissue by epigenetic modifications of genome [30, 31]; therefore, further study is necessary to analyze the transcriptome and epigenome of SA-β-Gal^+^ cells, to unravel the cellular and molecular mechanisms involved in long-term programming of cellular senescence as a result of PDE, and how a deregulation of cellular senescence retards long bone development in female mouse offspring.

## Conclusions

Although the precise mechanisms by which PDE enhances cellular senescence will require further experimentation, our studies have significant advancements by identifying cellular senescence as a key regulator linking PDE with postnatal bone development retardation in female mouse offspring, and further, targeting cellular senescence may be a promising avenue for prevention of bone development retardation in female mouse offspring after PDE.

## Supplementary information

**Additional file 1: Figure S1.** (a) Representative double staining images of senescence associated β-galactosidase (SA-β-Gal) and Nestin immunofluorescence. No much overlapping of SA-β-Gal^+^ and Nestin^+^ cells was observed. Scale bar, 100 μm. (b) Representative double staining images of senescence associated β-galactosidase (SA-β-Gal) and Osterix immunofluorescence. No much overlapping of SA-β-Gal^+^ and Osterix^+^ cells was observed. Scale bar, 100 μm.

**Additional file 2: Figure S2.** Representative double staining images of senescence associated β-galactosidase (SA-β-Gal) and Endomucin (Emcn) immunofluorescence. No much overlapping of SA-β-Gal^+^ and Emcn^+^ cells was observed. Scale bar, 100 μm.

## Data Availability

Please contact the corresponding author for data requests.

## Abbreviations

PDE: Prenatal dexamethasone exposure
GD: Gestational day
BMSCs: Bone marrow mesenchymal stem cells
SASP: Senescence-associated secretory phenotype
μCT: Microcomputed tomography
BV/TV: Bone volume
Tb. N: Trabecular number
Tb. Th: Trabecular thickness
Tb. Sp: Trabecular separation
H&E: Hematoxylin-eosin
TRAP: Tartrate-resistant acid phosphatase
SA-β-Gal: Senescence associated β-galactosidase
PBST: PBS with Tween
Emcn: Endomucin
ANOVA: Analysis of variance
D+Q: Dasatinib and quercetin
