# Supplementary figures and images for: Cellular senescence mediates the detrimental effect of prenatal dexamethasone exposure on postnatal long bone growth in mouse offspring
Source: Stem Cell Res Ther. 2020 Jul 6;11:270. doi: 10.1186/s13287-020-01790-9 (PMC7336470; doi:10.1186/s13287-020-01790-9)

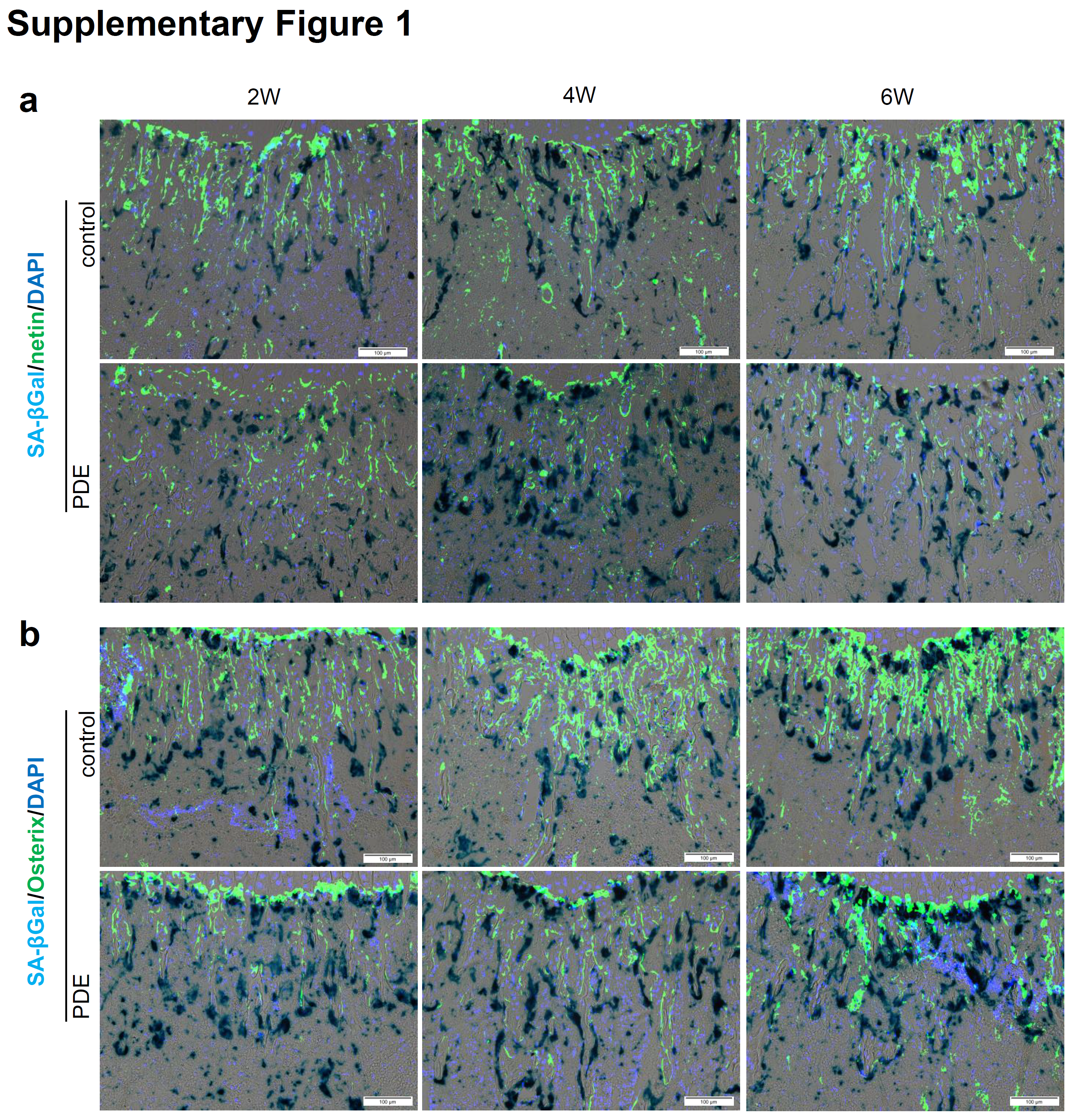

Supplement: Supplementary file 1 — Additional file 1: Figure S1. (a) Representative double staining images of senescence associated β-galactosidase (SA-β-Gal) and Nestin immunofluorescence. No much overlapping of SA-β-Gal+ and Nestin+ cells was observed. Scale bar, 100 μm. (b) Representative double staining images of senescence associated β-galactosidase (SA-β-Gal) and Osterix immunofluorescence. No much overlapping of SA-β-Gal+ and Osterix+ cells was observed. Scale bar, 100 μm. [file 13287_2020_1790_MOESM1_ESM.tif]

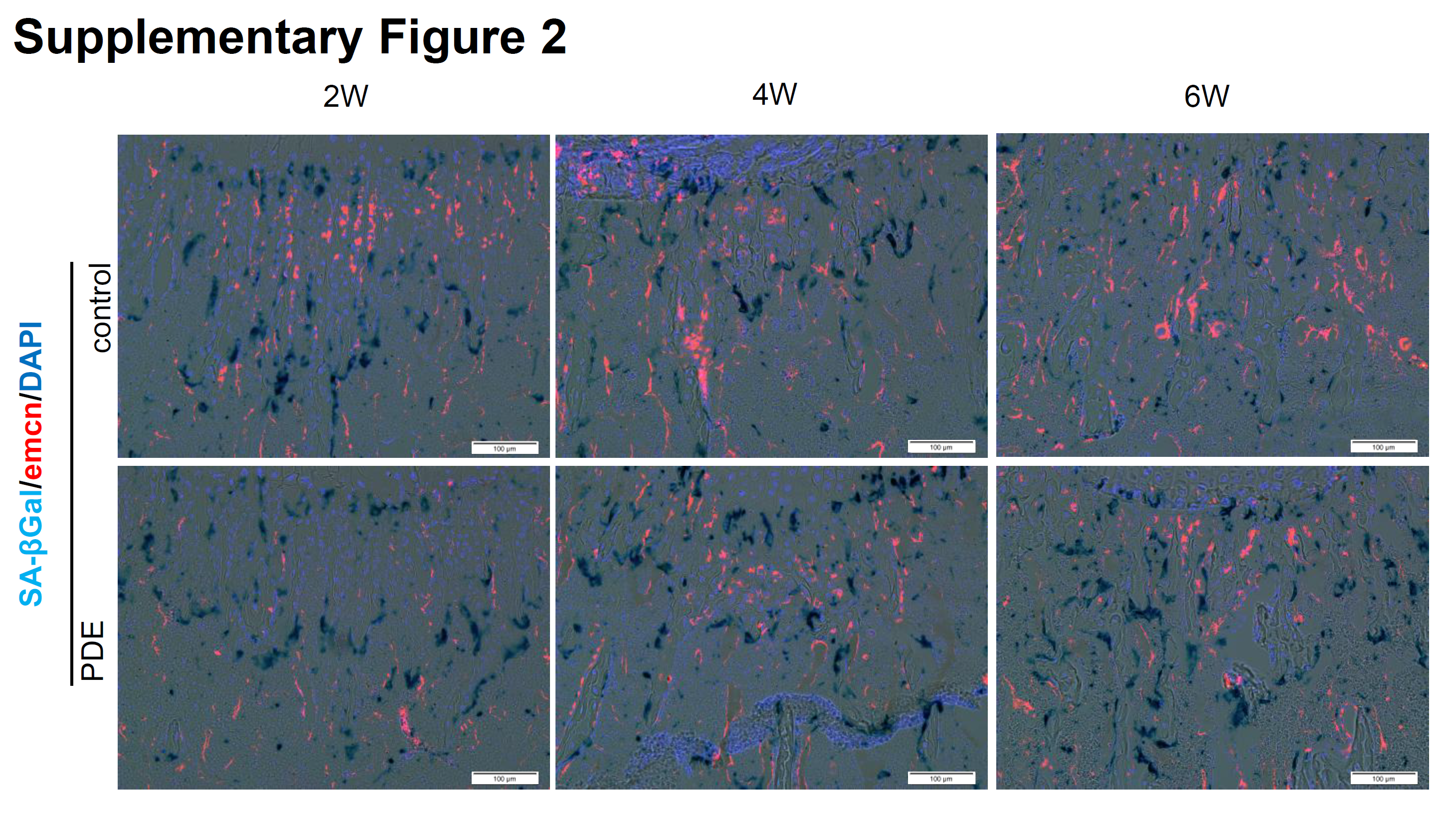

Supplement: Supplementary file 2 — Additional file 2: Figure S2. Representative double staining images of senescence associated β-galactosidase (SA-β-Gal) and Endomucin (Emcn) immunofluorescence. No much overlapping of SA-β-Gal+ and Emcn+ cells was observed. Scale bar, 100 μm. [file 13287_2020_1790_MOESM2_ESM.tif]
